# Peripheral Blood Mononuclear Cells Enhance Cartilage Repair in *in vivo* Osteochondral Defect Model

**DOI:** 10.1371/journal.pone.0133937

**Published:** 2015-08-07

**Authors:** Niina Hopper, John Wardale, Roger Brooks, Jonathan Power, Neil Rushton, Frances Henson

**Affiliations:** 1 Division of Trauma and Orthopaedic Surgery, University of Cambridge, Addenbrooke's Hospital, Cambridge, BC2 0QQ, the United Kingdom; 2 Department of Biological Sciences, University of Chester, Chester, CH1 4BJ, the United Kingdom; 3 Department of Veterinary Medicine, University of Cambridge, Cambridge, CB3 0ES, the United Kingdom; Rutgers - New Jersey Medical School, UNITED STATES

## Abstract

This study characterized peripheral blood mononuclear cells (PBMC) in terms of their potential in cartilage repair and investigated their ability to improve the healing in a pre-clinical large animal model. Human PBMCs were isolated with gradient centrifugation and adherent PBMC’s were evaluated for their ability to differentiate into adipogenic, chondrogenic and osteogenic lineages and also for their expression of musculoskeletal genes. The phenotype of the PBMCs was evaluated using Stro-1, CD34, CD44, CD45, CD90, CD106, CD105, CD146 and CD166 cell surface markers. Osteochondral defects were created in the medial femoral condyle (MFC) of 24 Welsh mountain sheep and evaluated at a six month time point. Four cell treatment groups were evaluated in combination with collagen-GAG-scaffold: (1) MSC alone; (2) MSCs and PBMCs at a ratio of 20:1; (3) MSCs and PBMC at a ratio of 2:1 and (4) PBMCs alone. Samples from the surgical site were evaluated for mechanical properties, ICRS score and histological repair. Fresh PBMC samples were 90% positive for hematopoietic cell surface markers and negative for the MSC antibody panel (<1%, p = 0.006). However, the adherent PBMC population expressed mesenchymal stem cell markers in hypoxic culture and lacked CD34/45 positive cells (<0.2%). This finding demonstrated that the adherent cells had acquired an MSC-like phenotype and transformed in hypoxia from their original hematopoietic lineage. Four key genes in muskuloskeletal biology were significantly upregulated in adherent PBMCs by hypoxia: BMP2 4.2-fold (p = 0.0007), BMP6 10.7-fold (p = 0.0004), GDF5 2.0-fold (p = 0.002) and COL1 5.0-fold (p = 0.046). The monolayer multilineage analysis confirmed the trilineage mesenchymal potential of the adherent PBMCs. PBMC cell therapy was equally good as bone marrow MSC therapy for defects in the ovine large animal model. Our results show that PBMCs support cartilage healing and oxygen tension of the environment was found to have a key effect on the derivation of a novel adherent cell population with an MSC-like phenotype. This study presents a novel and easily attainable point-of-care cell therapy with PBMCs to treat osteochondral defects in the knee avoiding any cell manipulations outside the surgical room.

## Introduction

Articular cartilage has a very limited capacity to repair. Defects greater than 3 mm are known to heal poorly with the formation of inferior fibrous cartilage [1, 2] and many attempts have been made to find the ideal treatment for large cartilage lesions. One of the major problems in cartilage healing is that lack of functional stem/progenitor cells in the tissue. In the absence of these endogenous stem cells, an alternative source of repair cells needs to be mobilised to heal cartilage lesions.

It is well known that a primitive cell population derived from circulating peripheral blood mononuclear cells (PBMC) can participate in the normal tissue renewal of various organs [3–7]. Unlike the majority of tissues, cartilage healing does not involve any direct mononuclear cell involvement as it is avascular, a consequence of which is that the tissue is hypoxic [8]. Osteochondral defect site is also relatively hypoxic at least until new blood vessels have developed into the repair tissue.

Cell populations present within PBMCs include CD14+ monocytes which originate from hematopoietic stem cells in the bone marrow and consist of 5 to 10% of circulating white blood cells in humans. They are committed cells derived from hematopoietic stem cells and a population of phagocyte precursors in transit from the bone marrow to their ultimate sites of activity in the tissues [9]. Monocytes are known to differentiate into several distinct phagocytes, including macrophages, dendritic cells (DS), osteoclasts, Kupffer cells, and microglia [9–12]. Current findings however, suggest that mononuclear cells have the potential to differentiate into cell types other than phagocytes, including bone, cartilage, fat, and skeletal and cardiac muscles [4, 6], making them potential candidate repair cells for cartilage.

Little is known about the effect of low oxygen tension on PBMCs. Peripheral blood monocytes are known to migrate and accumulate in hypoxic areas of inflammatory and tumour lesions [13]. MSCs derived from peripheral blood have been evaluated looking at the effect of hypoxia and serum deprivation in rabbit model [14] where the proliferation and apoptosis of peripheral blood MSCs was reported similar to bone marrow derived MSCs. Autologous mononuclear cells derived from bone marrow have also been tested in a rabbit model to heal full-thickness articular cartilage defects [15] [16] and their use has been compared to peripheral blood-derived mononuclear cells in rat [17], rabbit [18], sheep [19] and goat [20].

In the clinics peripheral blood mononuclear cell therapy has been used after arthroscopic subchondral drilling followed by postoperative intra-articular injections of autologous PBMCs in combination with hyaluronic acid (HA) in a clinical case study of 5 patients [21] and in a randomized controlled trial of 180 patients [22]. Another case series of 5 patients with early OA knee disease reported the use of intra-articular autologous PBSC injections in combination with growth factor addition/preservation (GFAP) and HA [23]. In addition, good clinical results have been reported with the use of PBMCs in the repair of large full-thickness cartilage defect combined with patellofemoral realignment in an autologous periosteum flap transplantation [24].

Various strategies can be employed to deliver cells to osteochondral defects *in vivo*, including being applied under membranes [25] and on scaffolds [26]. Work in our group has shown that a collagen/glycosaminoglycan biphasic scaffold can be used to support healing in an ovine osteochondral defect model *in vivo* [27]. This scaffold has been shown to support healing utilizing bone marrow derived cell therapy [28], and provides a model system in which to test the effects of PBMC on cartilage repair *in vivo*.

The aims of this paper were to characterize the effects of hypoxia on PBMC and to evaluate whether PBMC could be used for cartilage repair.

## Materials and Methods

### Mononuclear Cell Preparation

Peripheral blood mononuclear cells were prepared using fresh whole blood from 12 young (32.9 ± 9.3) healthy volunteers (4 female and 8 male donors) with full ethical consent in writing. The study was approved by the National Research Ethics Service Committee East of England Cambridge Central 06/Q0108/213. Blood was collected in Monovette EDTA tubes (Sarstedt) and a PBMCs prepared by density gradient centrifugation using Lymphoprep (Axis Shield) according to the manufacturer’s instructions. Mononuclear cells were either used fresh in the experiments at this stage or left to adhere and grow on a cell culture plastic. In some *in vitro* experiments PBMCs were also prepared from whole blood (NC13, 500 ml) and buffy coat (NC07, 60 ml) obtained from NHS Blood and Transplant Service, Cambridge.

### Cell culture

Standard cell culture conditions comprised a humidified atmosphere containing 5% CO2 in air (~20% O2). In order to mimic oxygen tension of hypoxic tissues, an environment comprising 90% nitrogen, 5% oxygen, 5% CO2 was achieved using a hypoxia controller unit (Proox model C21, BioSpherix, NY, USA) situated in a cell culture incubator. Unless otherwise stated, the cultures were under normal atmospheric oxygen tension (~20%) and grown in monolayers in Dulbecco’s Modified Essential Medium (DMEM) containing 10% foetal calf serum unless otherwise stated.

A biphasic collagen-glycosaminoglycan (GAG) scaffold (ChondroMimetic, Tigenix) was used for 3D cell culture studies and in the *in vivo* osteochondral repair model. ChondroMimetic comprised an unmineralised top layer mimicking articular cartilage while the base layer was mineralised using calcium phosphate (brushite) [29–31]. The material was cross-linked to enhance its mechanical strength and aimed to provide a similar microenvironment to cartilage. ChondroMimetic was soaked in culture medium for at least 12 h at 37°C before being seeded with the cells. In the *in vitro* experiments a total of 2.0 x 10⁵ cells were seeded per scaffold.

### Cell Phenotyping

Flow cytometry was used to characterise PBMCs for CD34, CD45, CD90 and CD105 cell surface markers using IM1839U, A07414, IM1870 and A07782 (Beckman Coulter) together with MSC antibody panel SC017 from R&D Systems (Stro-1, CD44, CD90, CD106, CD105, CD146 and CD166) according to the manufacturer’s instructions. Cells were analysed using a Beckman Coulter Cytomics FC500 flow cytometer instrument and the data was assessed with Kaluza Analysis Software. Positivity for each antibody was defined as the level of fluorescence >99% of the isotype-matched control.

Antibody-conjugated paramagnetic microbeads were used for a cell selection; CD14 Microbeads (130 050 200), CD105 Microbeads (130 051 200) and Monocyte isolation kit II (130 091 183) from Miltenyi Biotec and VersaLyse red blood cell lysing solution (A09777, Beckman Coulter) according to the manufacturer’s instructions. A heterogeneous mixture of peripheral blood mononucleated cells 2.0 x 10⁶/ml in PBS was discriminated using a FACSAria III (BD Biosciences, US) analyser according to light scatter signals FSC and SSC into monocytes, lymphocytes and granulocytes. Cell populations were analysed after normoxic and hypoxic culture conditions.

### Gene Expression

Total RNA was extracted using TRIzol reagent (15596–026, Ambion) according to the manufacturer’s instructions. The mRNA pellet was air-dried and resuspended in 35 μl DNAse/RNAse-free water, subsequently, concentration and quality were checked by OD 260/280 measurement with NanoDrop Spectrophotometer and 1.2% agarose gel electrophoresis using FlashGel System (57067, Lonza, US) and RNA Cassettes (57027, Lonza, US).

Complementary DNA synthesis was performed with SuperScript VILO kit (11754–050, Invitrogen) according to the manufacturer’s instructions. Real time quantitative PCR was prepared using QuantiFast SYBR Green PCR detection kit (204054, Qiagen) and quantified with a Stratagene Mx3000P real-time cycler using QuantiTect Primer Assays (Hs HIF1A 1 SG, Hs SOX9 1 SG, Hs BMP2 1 SG, Hs BMP6 1 SG, Hs GDF5 1 SG and Hs COL1A2 1 SG all from Qiagen).

### Multilineage Analysis

Adherent PBMCs were cultured as a monolayer with 1.5 x 10⁵ cells per well on a 12-well plate for 21 days in both normoxia and hypoxia. After confluency, the cells were treated with three differentiation media in triplicates for osteogenic, adipogenic and chondrogenic differentiation with basic medium used as a negative control. At the end of the experiment the cells were fixed, stained and analysed under a light microscopy.

For osteogenic differentiation [32–34] the medium consisted of 50 μg/mL L-ascorbic acid 2-phosphate (A8960-5G, Sigma), 10 mM β-glycerol phosphate (G9422-10G, Sigma), and 10 nM dexamethasone (50-02-2, Sigma). The medium was changed every 3–4 days for 21 days. At the end of the experiment the osteogenic cultures were fixed in 70% ethanol on ice and then stained with 2.0% alizarin red solution.

To promote chondrogenic differentiation StemPro Chondrogenesis Supplement (A10064-10, Invitrogen) was added to the basal medium and the medium was changed every 3–4 days for 21 days [35]. Chondrogenic cultures were fixed with acetone/methanol (1:1) and stained with 0.5% alcian blue (pH 0.75).

For adipogenic differentiation the StemPro Adipogenesis Supplement (A10065-01 Invitrogen) was added to the StemPro basal medium and changed every 3–4 days over a period of 21 days. The adipogenic cultures were fixed in 4% paraformaldehyde and then incubated with 60% isopropanol. Subsequently, the cultures were stained with fresh oil red O solution (three parts 0.3% in isopropanol with two parts water).

### Pre-clinical large animal model

All surgery was done in accordance with the regulations laid out in the Animals (Scientific Procedures) Act 1986 following UK Home Office and University of Cambridge Ethics Committee approval. A total of 24 skeletally mature female Welsh Mountain sheep (3–5 year old) were used. Each treatment group contained six sheep (n = 6). Four cell treatment groups were evaluated in combination with ChondroMimetic scaffold: (1) MSC alone; (2) MSCs and PBMCs at a ratio of 20:1; (3) MSCs and PBMC at a ratio of 2:1 and (4) PBMCs alone. ChondroMimetic scaffold (size 6.5 x 8.0 mm) was soaked in culture medium for 48 h at 37°C and a total of 1x10e6 ovine bone marrow derived MSCs (Mesoblast Ltd) were seeded 24 h prior to the surgery. Autologous PBMCs were isolated at the day of the operation and added to the scaffold during the surgery. The surgical technique was as described previously [27]. In a previous pilot study published by our research group [36] empty defect control and scaffold-alone controls were examined in a similar ovine osteochondral injury model together with a ChondroMimetic biphasic scaffold treatment. As no significant improvement was observed with the scaffold alone and MSC alone treatments compared to the empty defect, it was decided not to repeat these controls in this study in order to reduce the number of animals used. Briefly, full thickness osteochondral defects 6.0 mm diameter, 8 mm deep were created in the medial femoral condyle (MFC) via a medial parapatellar approach. General anaesthesia was induced with an injection of thiopentone (3 mg/kg) into the external jugular vein. Maintenance was achieved via inhalational anaesthetic of a mixture of isofluorane, nitrous oxide and oxygen. Perioperative analgesia was provided by pre-operative intramuscular Carprofen (1.5 mg/ml) and antibiotic prophylaxis was also given via intramuscular procaine penicillin (10 mg/ml). Postoperatively, animals were allowed to fully weight bear. Animals were humanely sacrificed at 26 weeks postoperatively using a lethal dose of sodium pentobarbital.

### Gross Morphology

At post-mortem, joints were blindly scored using the International Cartilage Repair Society (ICRS) score [37] to assess the integration of the scaffold into the joint [38].

### Mechanical Testing

Stiffness measurements were taken from the centre of the osteochondral defect and at a distance of 1 mm from the original edge of the created osteochondral defect at the 12-, 3-, 6-, and 9-o’clock positions and 1 mm from the edge in the perilesional cartilage, using a handheld digital durometer (Shore S1, M scale, Instron, Norwood, MA). A number between 0 and 100 was given with a built-in calibrated error of five. These measurements were then repeated in the contralateral limb in the same anatomic sites. The stiffness of the reparative tissue was then expressed as a percentage of stiffness relative to the control cartilage of the contralateral limb.

### Histology

The tissue specimens were harvested, snap-frozen in liquid nitrogen and then stored in -80°C. Tissue was embedded in OCT Cryoembedding Compound (SDLAMB/OCT, Fisher) and sections of 10 μm thickness were made through the central portion of the defect. Sections were stained with Safranin O/Fast Green, anti-human collagen type I and type II mouse monoclonal antibody (08631701 and 08631711, MP BIOMEDICALS) and blindly scored using a modified O’Driscoll score as a guide [39–43].

### Data Analysis

All samples were collected as four replicates and the data is presented as the mean ± standard deviation (SD) unless otherwise stated. The results of quantitative RT-PCR were analysed by the ΔΔCt method using online software (Qiagen). All other results were analysed using IBM SPSS statistics version 22; this showed non-homogeneity of variance using Levene’s test and groups were therefore examined using the Kruskal-Wallis test for significant differences, the Shapiro-Wilk test for normal distribution, which showed data sets were normally distributed, and finally by Games-Howell post-hoc testing. The significance level was set at 0.05.

## Results

### Cell phenotyping

**Fig 1:**
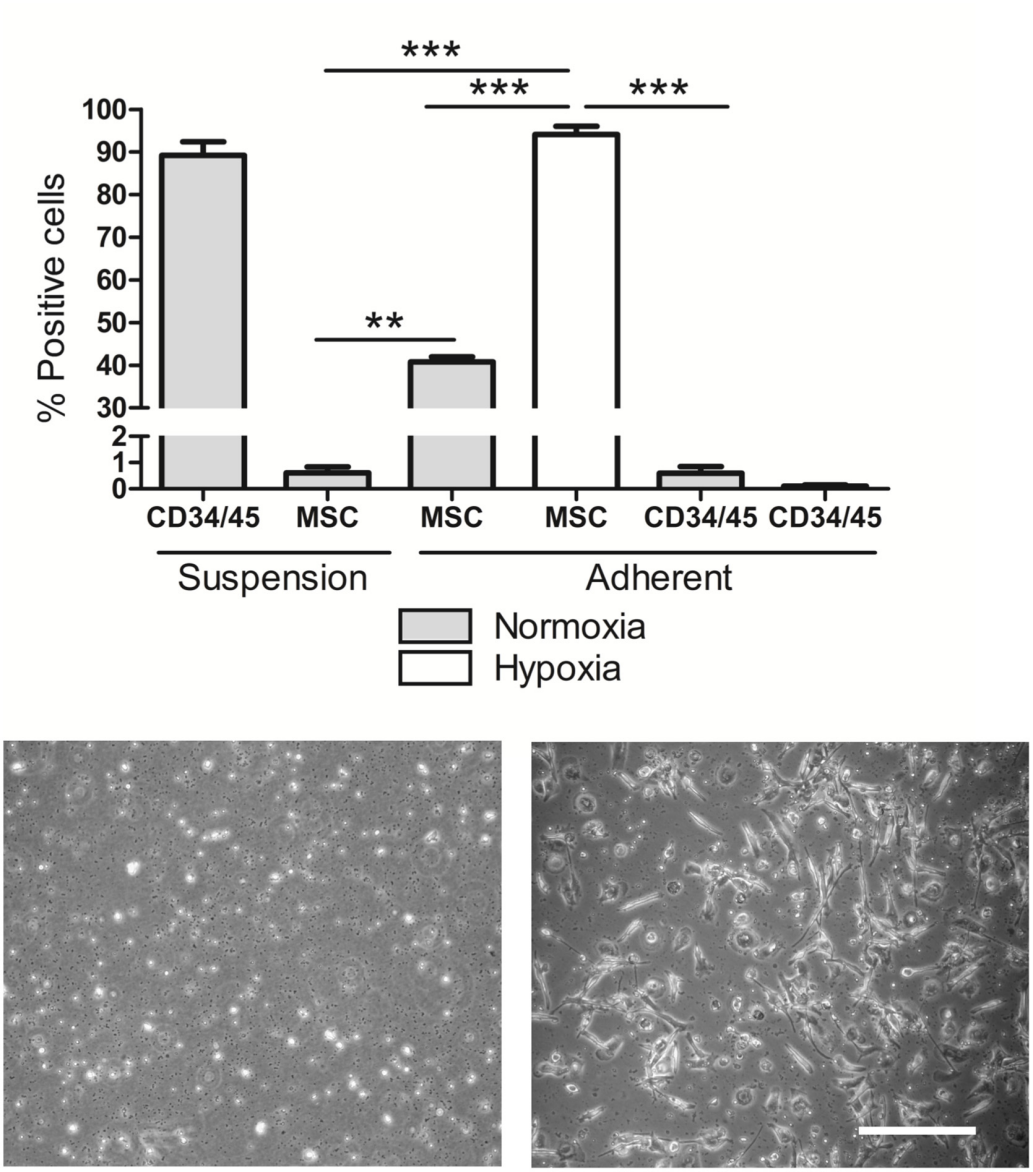
Peripheral blood mononuclear cell characterization. (A) Fluorescent labelling of fresh PBMC in suspension and adherent PBMC in both normoxia and hypoxia comparing hematopoietic and mesenchymal cell surface markers (n = 4). Representative images of PBMCs after 12 days growing in (B) normoxia and (C) hypoxia (scale bar 50 μm).

The fresh PBMC samples were 90% positive for the hematopoietic cell surface markers CD34/45 (combined) and negative for the MSC antibody panel (<1.0%, p = 0.006) (Fig 1A). After 2 weeks of hypoxic culture adherent peripheral blood mononucleated cell population had a fibroblast-like morphology (Fig 1C) and 94% expressed mesenchymal stem cell markers; Stro-1, CD90, CD106, CD105, CD146, CD166 and CD44) (p<0.0001) compared to 41% in normoxia where cells were more rounded. At the same time as the PBMCs had become adherent and changed their phenotype to MSC positive there was a concomitant decrease in hematopoietic phenotype markers CD34/45 (<0.2%, p = 0.0008). These findings demonstrated that the adherent cells had acquired an MSC-like phenotype and transformed from their original hematopoietic lineage.

The mononuclear cells from whole peripheral blood were analyzed with magnetic labeling, fluorescence assisted cell sorting and cell sorting. After 2 weeks in low oxygen (5%) *in vitro* culture, PBMCs produced a cell population with a fibroblast-like morphology (Fig 1C). Under normoxic culture conditions, an adherent cell population attached to the cell culture plastic (Fig 1B) eventually transforming into macrophages.

Whole blood samples separated with magnetically labeled microbeads into monocytes, CD14 and CD105 positive cells or by cell sorting separating into lymphocytes 73.7%, monocytes 1.9% and granulocytes 0.9% did not produce adherent fibroblast-like cell populations after 30 days in normoxic or hypoxic cultures but transformed into macrophage-like adherent cells. It was concluded that individual cell populations did not yield the adherent fibroblast-like cell type as seen in the heterogeneous whole PBMC culture in hypoxia.

### Cartilage microenvironment and gene expression

**Fig 2:**
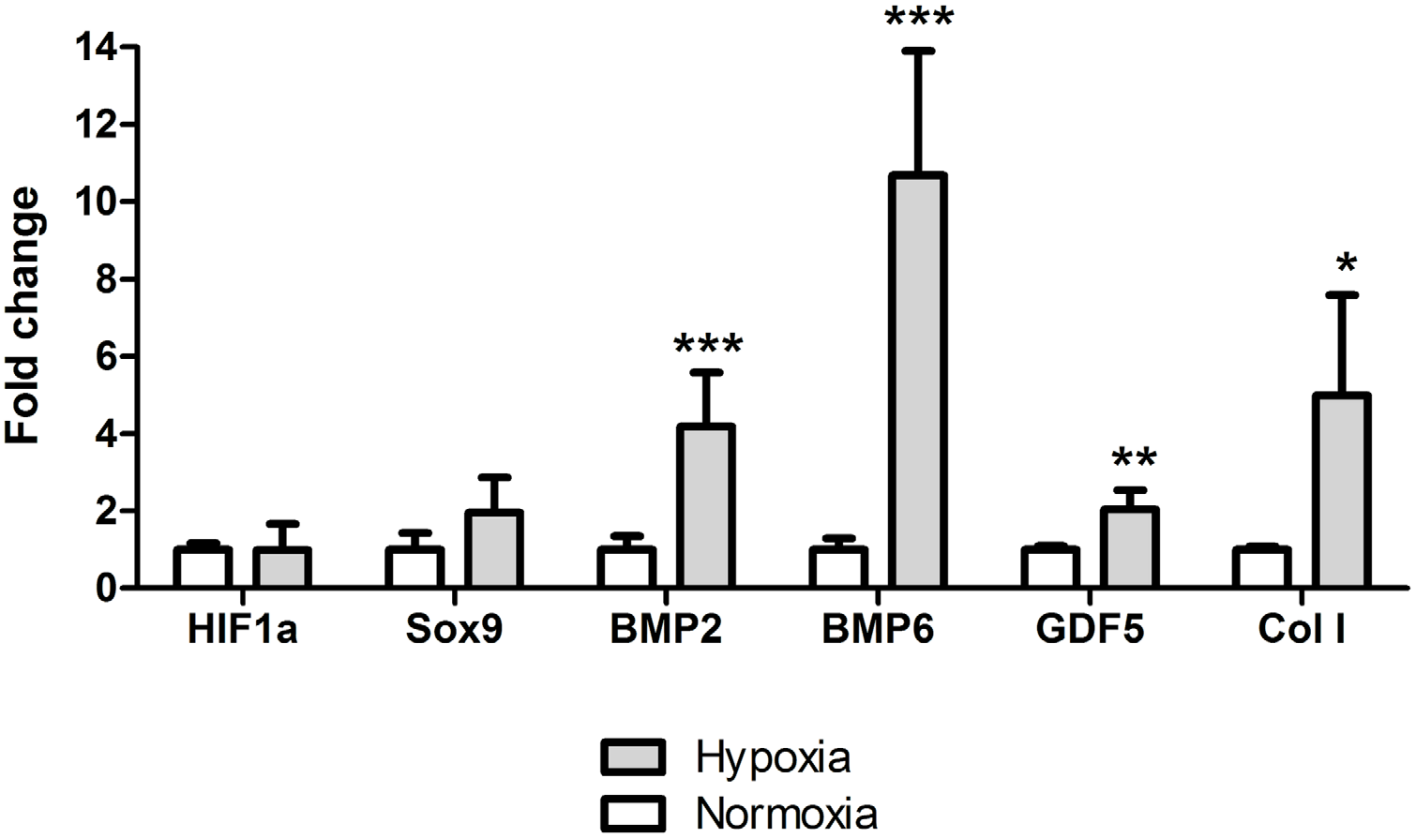
Gene expression analysis. The mRNA expression of PBMCs in both normoxic and hypoxic culture (24h). BMP2 (p = 0.0007), BMP6 (p = 0.0004), GDF5 (p = 0.002) and COL1 (p = 0.046) normalized to B2M housekeeping gene. Level of statistical significance; * p<0.05, ** p<0.001 and *** p<0.0001 with biological n = 4 and technical n = 3.

The fold regulation of HIF1α, SOX9, BMP2, BMP6, GDF5 and COL1 in both normoxia and hypoxia was normalized to B2M housekeeping gene which was found stable under reduced oxygen tension. Of these six genes, four were significantly upregulated in PBMCs by the reduced oxygen tension at 24 h; BMP2 4.2-fold (p = 0.0007), BMP6 10.7-fold (p = 0.0004), GDF5 2.0-fold (p = 0.002) and COL1 5.0-fold (p = 0.046) Fig 2A. HIF1α is a master regulator when cells are adopting to lowered oxygen tension, however, since HIF1α is mainly regulated post-translationally, therefore the gene expression cannot be used as a marker for hypoxic environment [44].

### Multilineage analysis

**Fig 3:**
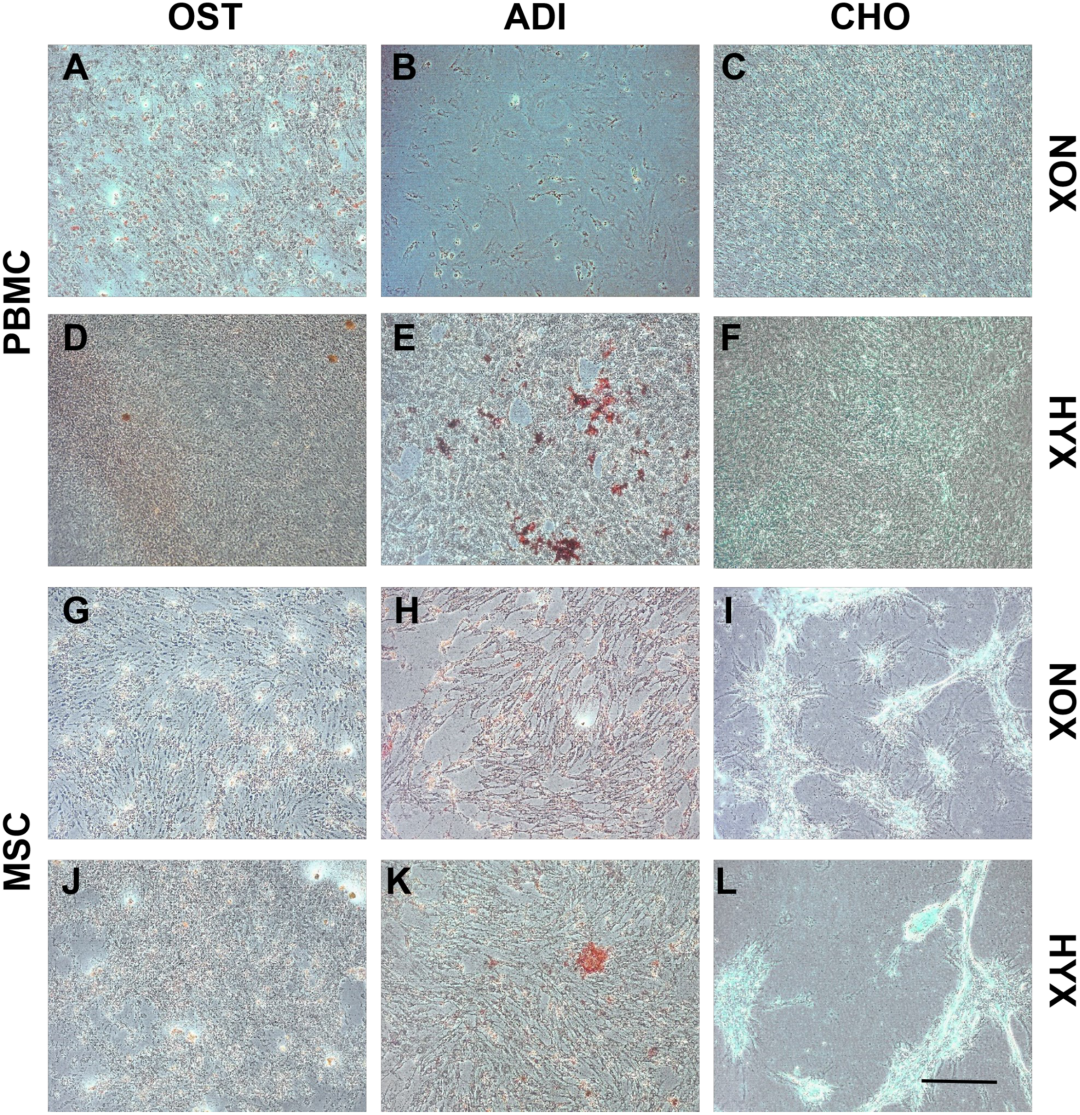
Tripotential lineage differentiation. Morphological analysis of the adherent PBMCs (A-F) and Mesoblast MSCs (G-L) in both normoxic and hypoxic culture at day 21 under phase contrast light microscopy. Representative images of osteogenic differentiation (Alizarin-red; A, D, G and J), adipogenic differentiation (Oil-red-O; B, E, H and K) and chondrogenic differentiation (Alcian blue; C, F, I and L). Scale bar 200 μm.

By day 10 all hypoxic cell cultures had reached confluency with those cultured in normal oxygen tension remaining subconfluent and eventually differentiating into macrophage-like cells. Adherent mononucleated cells in hypoxia treated with the osteogenic differentiation medium underwent a change in their morphology from spindle-shaped to cuboidal and formed calcium deposits in culture (Fig 3A). Following the adipogenic differentiation treatment, lipid vacuoles stained positive with oil red O (Fig 3B). Chondrogenic differentiation induced a change in the PBMC morphology, with alcian blue staining confirming aggregated areas positive for glycosaminoglycans (Fig 3C).

### Large animal model

#### Surgical Observations at Implantation

The operations were uneventful and all animals recovered from surgery without incident. The incisions closed successfully and there were no incidences of infection. All animals demonstrated normal weight gain and maintenance indicative of normal weight-bearing. None of the 24 animals had to be excluded from the study.

#### Gross Morphological Findings

**Fig 4:**
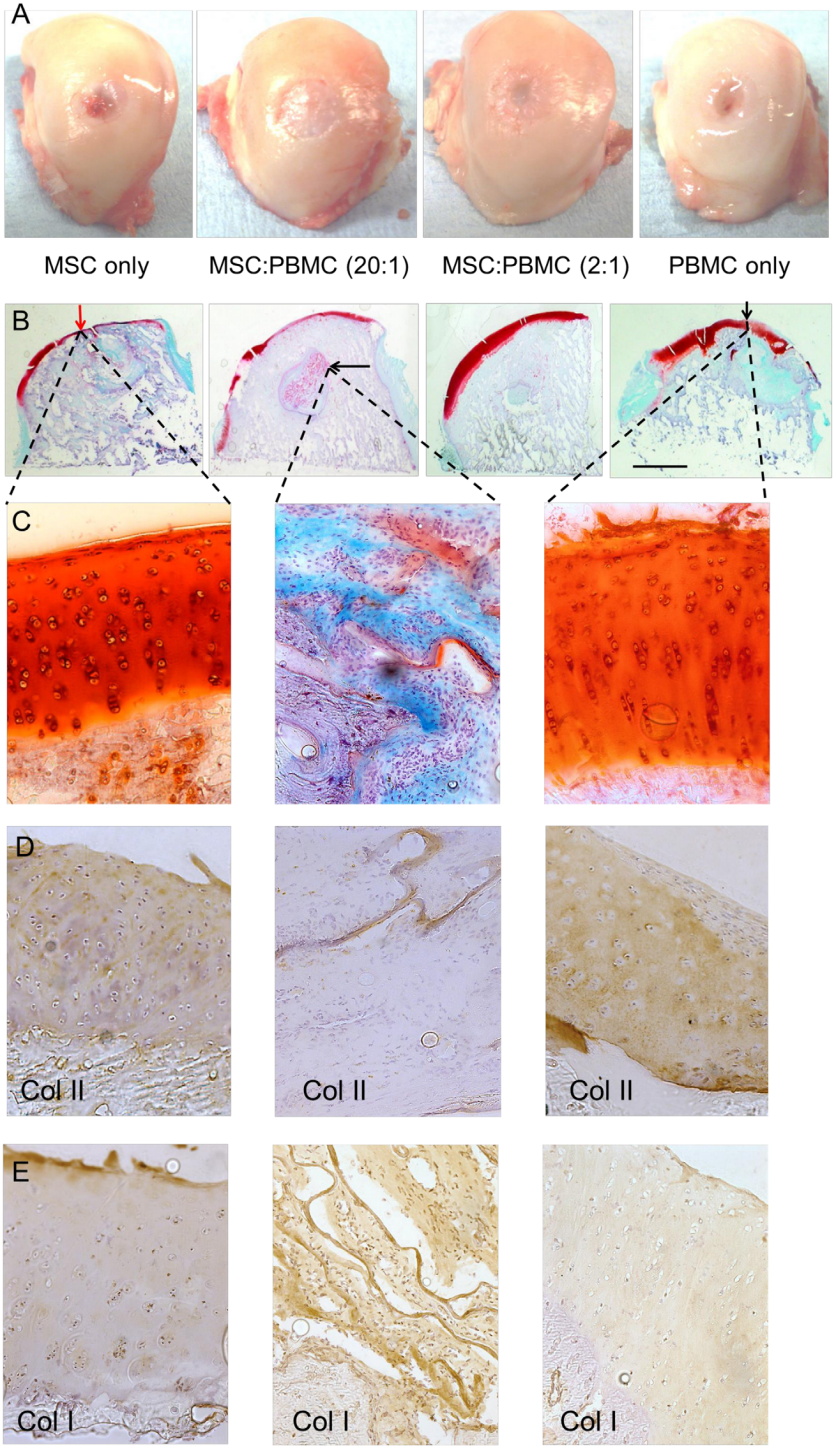
Analysis of the defect repair. (A) Representative images of an average sample of each treatment group showing the macroscopic surface repair in femoral condyles. (B) Osteochondral healing of each treatment group stained with Safranin O/Fast Green. The scale bar represents the radius of the initial defect (6.0 mm). Some of the findings include: neocartilage formation on the surface of the defect (red/black vertical arrow) and remnants of the biomaterial (black horizontal arrow). (C) Safranin O/Fast Green stained high magnification (20x) images of the articular cartilage healing in the surface and in the subchondral bone where remnants of the biomaterial can be found. (D) Collagen type II staining and (E) Collagen type I staining at the repair site and within the remnants of the collagen biomaterial.

Most samples had good macroscopic surface repair with good integration with only a minimal chronic inflammatory response. Macroscopic repair was not fully matured at 26 weeks, although all samples demonstrated substantial amounts of defect closure and a large proportion had produced smooth hyaline-like articular cartilage at the defect site (Fig 4A). In some cases there were visual indications of the scaffold location based on variations in opacity and non-uniform surface features associated with incomplete fusion of the cartilage layer. No significant functional differences were found between operated sites compared to contralateral controls.

**Fig 5:**
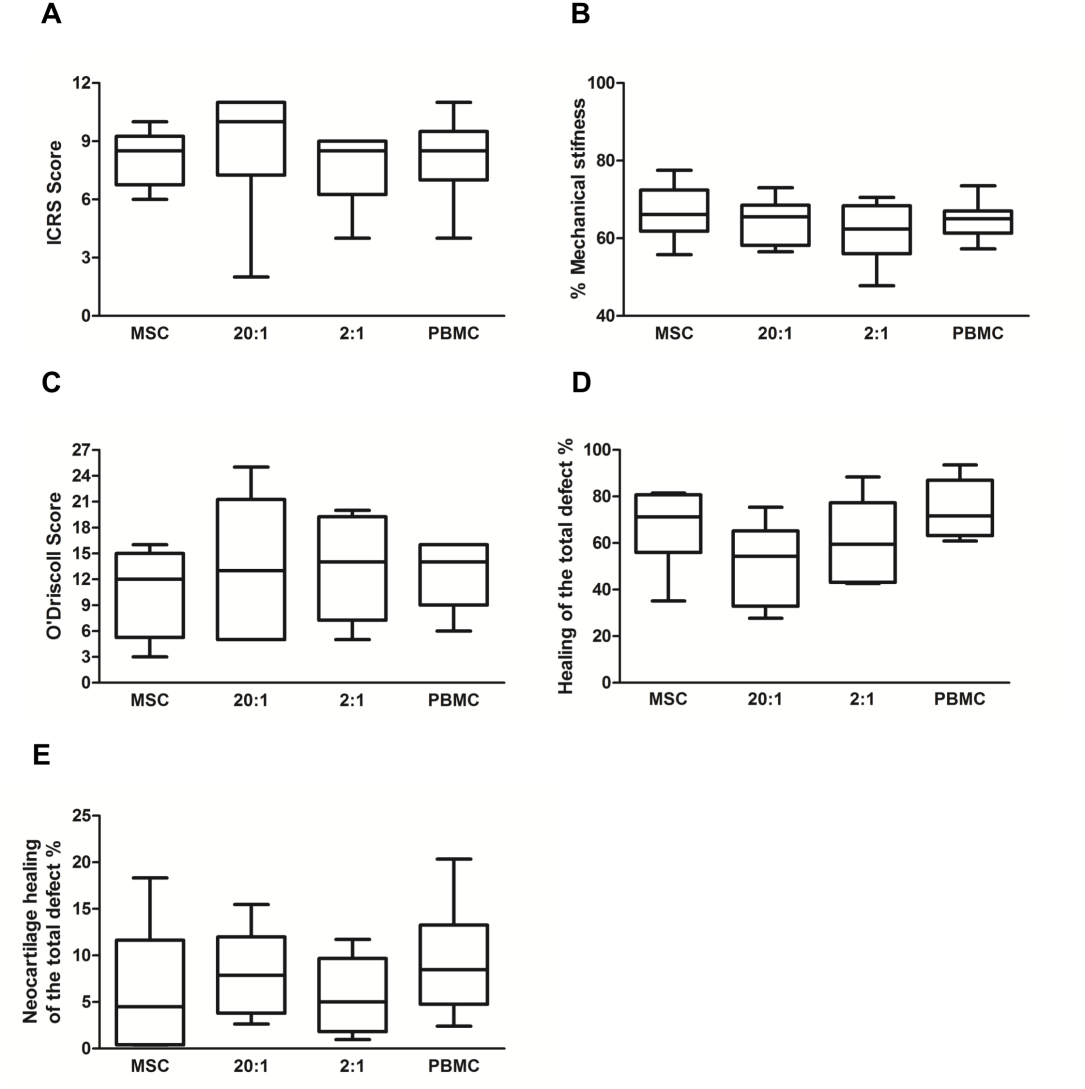
Quantification of the repair tissue. (A) The ICRS score assessing the integration of the cell-scaffold construct into the medial femoral condyles. (B) Mechanical stiffness and (C) histological evaluation based on the modified O'Driscoll scoring system. (D) Summary of the healing with repair tissue in the defect when 100% is the total defect area. (E) Summary of the neocartilage formation in the articular cartilage surface when 100% is the total defect area. There was no significant difference between the test groups for these measurements.

The ICRS macroscopic score assessing the integration of the cell-scaffold construct into the defect site presented no significant differences between the MSC and PBMC treatment groups (Fig 4B). Every cell therapy treatment group presented early normal cartilage repair and averaged scores between 8 and 9 (Fig 5A).

#### Histological Findings

More than 50% of the total defect area had healed during the 26 weeks in all test groups (Fig 5D) as quantified by the Safranin O/Fast Green staining (Fig 4B and 4C) whilst the remaining area showed healing still in progress. In most cases the surface had healed with hyaline neocartilage tissue (Fig 4D) with occasional remnants of the scaffold only observed deep in the defect area. The majority of the treatment groups presented excellent restoration of the subchondral bone plate. Tissue morphology in the PBMCs 2:1 and PBMC only treatment groups demonstrated early normal hyaline cartilage with nearly complete thickness (Fig 4B). There was no significant difference in the healing between MSC and PBMC only cell treatment groups even though PBMC therapy had slightly higher histological repair scores than MSC treatment (Fig 5). Analysis using the modified O’Driscoll score (Fig 5C) demonstrated that the greatest repair activity within the original defect area including matrix deposition, hyaline cartilage thickness and bonding to adjacent tissue was observed with the PBMC treated defects. The addition of PBMCs increased neocartilage formation in every treatment group as compared to the MSC alone treatment (Fig 5E). The increase in the neocartilage formation was greatest (62.5±7.9%) in the PBMC alone treatment as compared to the MSC alone as quantified from the total defect area (Fig 5E). Most healing was detected consistently in the treatment groups where PBMCs were added as measured with ICRS score, O’Driscoll score, total healing and neocartilage formation.

## Discussion

In this paper we have demonstrated, for the first time, that hypoxia drives the differentiation of PBMC into MSCs and that these PBMC-derived MSCs are functional and capable of inducing cartilage repair *in vivo*.

Culturing PBMCs in monolayer resulted in an adherent cell population that was 40% positive for markers of an MSC phenotype. Hypoxia had a profound effect on these PBMC cultures increasing the proportion of cells expressing MSC markers in the population to 94% (p<0.0001). This transformation was concomitant with a loss of markers for the hematopoietic cell origin (CD34/45, p = 0.0008). Interestingly, hypoxia preconditioning has been reported to have a supporting effect on bone marrow MSC cell therapy applications in the liver [45, 46] and cartilage [47].

Based on our study, the key to the biological mechanism triggering the adherent mononuclear cell selection is reduced oxygen tension of the target tissue. These observations might explain some of the complex roles of mononucleated cells. PBMCs circulate in the peripheral blood in high oxygen tension expressing hematopoietic markers and have the potential to migrate into the target tissue (reduced oxygen tension) and to transform into macrophages, denritic cells, or fibrocytes [3, 48–50]. Together with chemical signaling molecules oxygen tension may be one of the indicators stimulating mononuclear transformation.

From our findings, it is possible that mononuclear cells in peripheral blood are not solely phagocyte precursors but multipotent precursors or a group of monopotent precursors for several distinct lineages, including non-hematopoietic cells, which can differentiate independently into their corresponding mature tissues. Evidence that these adherent PBMCs are multipotent, rather than being a mixture of committed progenitor cells each with a restricted potential, includes their characteristic morphology, the presence of typical protein markers, and their differentiation into mesenchymal lineages. This hypothesis was also supported by a wound healing model in murine using peripheral blood stem cells together with TGF-β and FGF growth factors [51].

Since peripheral blood mononucleated cells are a heterogeneous mixture of cells, we used cell sorting to produce individual cell populations in an attempt to isolate a cell type that was the parent population to the adherent PBMC. Isolation of CD14 and CD105 cell populations or sorting the PBMC sample into lymphocyte, monocyte and granulocyte populations both failed to produce an adherent fibroblast-like cell population. This suggests that in a heterogeneous mixture of cells [52], cell-cell contact [53], and cell signaling [54] may be required for the adherent cell type to mature and transform [55] as demonstrated in stem cell co-culture models [56]. This experiment rules out the possibility that the origin of the adherent cells is due to a specific type of cell that is present in low numbers in the original blood.

To test the potential of the PBMC-derived MSCs for cartilage repair, chondrogenic gene expression in both normoxia and hypoxia was measured *in vitro*. Four key genes in musculoskeletal repair were significantly upregulated in PBMCs by the reduced oxygen tension; BMP2, BMP6, GDF5 and COL1. The monolayer multilineage analysis confirmed the tripotential mesenchymal potential of the adherent PBMCs to differentiate into chondrogenic, osteogenic and adipogenic lineages similar to the report by Kuwana et al. (2003) [4]. This data clearly demonstrates the potential for PBMCs to generate cells that would express genes and proteins beneficial in musculoskeletal repair when placed in a hypoxic environment as found in an osteochondral defect.

Several clinical investigators from various parts of the world have reported on the safety and therapeutic effect of both autologous and allogeneic MSC transplantation in patients with osteoarthritis in the knee [57]. Because MSCs are generally hypoimmunogenic and possess immunosuppressive activity, therefore, the use of MSCs for allogeneic therapy does not require HLA matching [58]. As ‘proof of concept’ that PBMC-derived MSCs are functionally capable of inducing cartilage repair, we compared of bone marrow MSCs and peripheral blood PBMCs, delivered on a previously characterized biphasic scaffold [29–31] in a large animal osteochondral defect model [36]. In this model the MSCs and PBMCs showed similar healing capacities, with no adverse inflammatory reaction at the implantation site.

Our results together with previous reports demonstrate that peripheral blood derived mononuclear cells have similar properties in cartilage healing as compared to autologous mononuclear cells derived from bone marrow in rat [17], rabbit ([18], sheep [19] and goat [20]. To date, few publications have described the use of PBMCs to treat cartilage lesions with good clinical results. These published repair strategies include postoperative intra-articular injections of autologous PBMCs in combination with hyaluronic acid (HA) [21, 22], intra-articular autologous PBSC injections in combination with growth factor addition/preservation (GFAP) and HA [23] and PBMCs with an autologous periosteum flap transplantation [24].

## Conclusion

Our study has provided evidence that PBMC can be a source of cells to stimulate the healing in osteochondral lesions. The advantages of PBMC therapy includes the fact that in contrast to other sources of multipotent cells, the isolation of peripheral blood is minimally invasive and does not require general anaesthesia. Blood is the most convenient source from which to obtain PBMCs from patients, which can be frozen and stored for later use, can be obtained regardless of patient age, are easily derived in large quantities and when used as an autograft produces no rejection removing the need for immunosuppressive therapy and so can be used without ethical constraints [59]. PBMC-based cell therapies would avoid the need of a time-consuming and expensive manipulation of cells in laboratory cultures providing a simplified point of care solution to the operating surgeon. Thus, potential clinical applications of peripheral blood-derived stem cells are of great interest. Our findings, taken together with previous work, indicate that circulating mononuclear cells are more diverse than previously thought, making cell transplantation therapies using circulating mononucleated cells a potential approach for future tissue regeneration.
